# Lgr5 deficiency accelerates intervertebral disc degeneration via NF-κB activation and enhanced osteoclastogenesis

**DOI:** 10.3389/fmed.2026.1939627

**Published:** 2026-10-14

**Authors:** Feihong Che, Qiangqiang Zhao, Jiayang Wang, Guoli Huang, Lijuan Yu, Gang Li, Liangliang Xu, Ding Wang

**Affiliations:** 1 Lingnan Medical Research Center, The First Clinical Medical School of Guangzhou University of Chinese Medicine, Guangzhou University of Chinese Medicine, Guangzhou, China; 2 College of Artificial Intelligence, Shandong Vocational and Technical University of Engineering, Jinan, China; 3 School of Basic Medical Science, Guangzhou University of Chinese Medicine, Guangzhou, China; 4 Center for Locomotor System Regenerative Medicine and Technology, Institute of Biomedicine and Biotechnology, Shenzhen Institute of Advanced Technology, Chinese Academy of Sciences, University Town of Shenzhen, Shenzhen, China; 5 Guangdong Clinical Research Academy of Chinese Medicine, Guangzhou, China; 6 The Eighth Clinical Medical College of Guangzhou University of Chinese Medicine, Foshan Hospital of Traditional Chinese Medicine, Foshan, China

**Keywords:** inflammation, intervertebral disc degeneration, LGR5, NF-κB pathway, nucleus pulposus, osteoclast

## Abstract

**Background:**

Intervertebral disc degeneration (IDD) is a common degenerative disease and the primary cause of low back pain. Leucine-rich repeat-containing G-protein coupled receptor 5 (Lgr5) is an activator of the canonical Wnt signaling pathway. But its role in IDD progression and the underlying molecular mechanisms remain poorly understood.

**Methods:**

Lgr5 knockout (Lgr5 KO) mice were generated by crossing Lgr5^flox/flox^ mice with Prx1-Cre transgenic mice. These samples were analyzed by RNAseq, micro-CT, histology and western blotting, etc.

**Results:**

MicroCT analysis of Lgr5 KO and wild-type (WT) mice caudal vertebrae showed that Lgr5 KO mice exhibited narrowed disc space, deteriorated trabecular bone microarchitecture, and decreased bone volume fraction (BV/TV). In a surgically induced IDD model, we showed that Lgr5 KO led to disrupted disc structure, nucleus pulposus (NP) atrophy, cellular loss, and decreased glycosaminoglycan content, thereby accelerating the progression of IDD. RNA-seq analysis identified 900 differentially expressed genes (457 upregulated, 443 downregulated) in Lgr5 KO discs, enriched in osteoclast differentiation, ECM-receptor interaction, and NF-κB signaling pathways. TRAP staining showed enhanced osteoclast activity in Lgr5 KO endplates. Western blot demonstrated reduced Collagen II and increased MMP13, p-p65 in Lgr5 KO NP cells. Immunofluorescence and immunohistochemical staining confirmed that Lgr5 deficiency increased MMP13, p-p65, and Active caspase-3, while decreasing PCNA, promoting inflammation, apoptosis, and suppressing proliferation in NP cells.

**Conclusions:**

Collectively, our results demonstrated that Lgr5 deficiency increased inflammation, disrupted ECM homeostasis and NP cell functions, which finally accelerated IDD.

## 1 Introduction

Intervertebral disc degeneration (IDD) is the primary pathological basis of low back pain, a leading cause of global disability (1, 2). The degenerative cascade involves multiple interrelated processes, including extracellular matrix (ECM) dysregulation, excessive apoptosis of nucleus pulposus (NP) cells, chronic low-grade inflammation, and aberrant ossification of the cartilaginous endplates (3). As the global population ages, the prevalence of IDD continues to escalate, imposing substantial socioeconomic burdens and severely impairing quality of life (4, 5). Despite well-established contributory factors—including advanced age, genetic predisposition, biomechanical overload, and inflammatory mediators—the precise molecular pathogenesis of IDD remains incompletely understood (6, 7). Consequently, identifying key regulatory genes and signaling nodes that govern IDD progression is crucial for developing mechanism-based therapies.

Leucine-rich repeat-containing G-protein coupled receptor 5 (Lgr5), a canonical co-receptor of the Wnt/β-catenin signaling pathway, was initially identified as a specific marker of intestinal stem cells and has since been recognized as a master regulator of stem cell proliferation, differentiation, and tissue regeneration across multiple organ systems (8). Emerging evidence implicates Lgr5 in cartilage homeostasis, with studies demonstrating its ability to preserve the chondrocyte phenotype and mitigate osteoarthritis progression via the PI3K/AKT pathway (9). A recent groundbreaking work by Ruscitto et al. revealed that Lgr5-expressing secretory cells form a Wnt inhibitory niche in cartilage that is critical for maintaining chondrocyte identity, and that Lgr5 ablation during aging or osteoarthritis results in depletion of Wnt-inactive chondroprogenitors and a surge of Wnt-activated, phenotypically unstable chondrocytes (10, 11). However, the functional role of Lgr5 in IDD—particularly in the context of aging—and its downstream molecular effectors remain largely unexplored, representing a critical gap in our understanding of disc biology (12).

The NF-κB pathway, a master switch for inflammatory gene expression, drives the transcription of matrix metalloproteinases (MMPs), including MMP13, upon phosphorylation of its p65 subunit (p-p65) (13–15). Furthermore, osteoclast-mediated resorption and subsequent ossification of the cartilaginous endplate compromise nutrient diffusion to the avascular disc, accelerating degenerative changes (16, 17). Notably, activation of osteoclastogenic pathways has been directly implicated in endplate remodeling and IDD progression.

In the present study, we utilized 8-month-old Lgr5 KO mice generated by crossing Lgr5^flox/flox^ mice with Prx1-Cre transgenic mice to observe the effect of Lgr5 deletion on intervertebral disc degeneration (IDD). We demonstrate for the first time that loss of Lgr5 aggravates IDD by activating NF-κB signaling, promoting osteoclast activity, and disrupting nucleus pulposus (NP) cell homeostasis. These findings indicate that Lgr5 serves as a crucial protective regulator and represents a promising therapeutic target for alleviating intervertebral disc degeneration.

## 2 Materials and methods

### 2.1 Animal

The Prx1-Cre-mediated conditional Lgr5 knockout (Lgr5 KO) mice used in this study were generated by Cyagen Biosciences (Suzhou, China). All mice were purchased from the Center of Laboratory Animals, Guangzhou University of Chinese Medicine, and housed in a standard SPF environment under controlled conditions: temperature 22–25 °C, relative humidity 50%–60%, 12 h light/12 h dark cycle, with free access to standard rodent chow and filtered water.

### 2.2 IDD model

Eight-month-old Lgr5 KO mice and Lgr5^flox/flox^ mice were used to establish the surgically induced intervertebral disc degeneration (IDD) model (*n* = 10 per group). In parallel, age-matched naive mice of both genotypes (*n* = 5 per group) were maintained without surgery to assess baseline phenotypes. In brief, mice were anesthetized with Delivector™ Avertin (tribromoethanol, Dowobio, Shanghai) at a dosage of 250 mg/kg body weight via intraperitoneal injection. Then a 30-gauge needle was inserted into the caudal vertebra discs to penetrate the annulus fibrosus and nucleus pulposus, maintained for 10 s before slow withdrawal, with all surgeries conducted by a blinded investigator. Eight weeks after surgery, mice were euthanized via an overdose of Delivector™ Avertin at 500 mg/kg body weight via intraperitoneal injection, followed by cervical dislocation to confirm death.

### 2.3 RNA sequencing and bioinformatic analysis

Total RNA was extracted from intervertebral disc tissues using TRIzol reagent (Invitrogen, USA). RNA purity and integrity were verified via Nanodrop 2,000 (Thermo Fisher Scientific, USA) and Agilent 2,100 Bioanalyzer (Agilent Technologies, USA), respectively. cDNA libraries were constructed from three independent biological replicates per group (*n* = 3) and sequenced on an Illumina NovaSeq 6,000 platform (Illumina, USA). After quality control, differentially expressed genes (DEGs) were identified using DESeq2 with thresholds of |log2FC| ≥ 1 and adjusted *P* < 0.05. Gene Ontology (GO) and Kyoto Encyclopedia of Genes and Genomes (KEGG) enrichment analyses were performed to delineate biological processes and signaling pathways associated with DEGs.

### 2.4 Micro-computed tomography (micro-CT)

The age-matched naive mice spine specimens were fixed in 4% paraformaldehyde (PFA) for 24 h at 4 °C and scanned using a SkyScan 1,176 micro-CT system (Bruker, Belgium) at 50 kV, 500 μA, with an 18 μm isotropic voxel size. Three-dimensional reconstructions and quantitative analyses of disc height and trabecular bone volume fraction (BV/TV) were performed using CTAn software (Bruker, Belgium). Three independent specimens per group were analyzed in triplicate.

### 2.5 Histological staining

Following decalcification in 10% EDTA (pH 7.4) and paraffin embedding, 5 μm-thick sagittal serial sections were subjected to hematoxylin and eosin (H&E) staining for general morphology, Safranin O/Fast Green (SFO) staining for glycosaminoglycan (GAG) content, and TRAP staining (Sigma-Aldrich, USA) for osteoclast identification, performed according to the manufacturer's instructions. TRAP-positive areas (purplish-red) and cell counts were quantified using ImageJ software (NIH, USA) from five random high-power fields (200×) per section. For osteoclast marker validation, immunohistochemical staining for CTSK (Abcam, ab19027, rabbit polyclonal, 1:200) and c-Fos (Abcam, ab222699, rabbit monoclonal, 1:500) was performed on adjacent sections.

### 2.6 Nucleus pulposus cell isolation and culture

NP tissue was microdissected from caudal vertebral discs under sterile conditions and digested sequentially with 0.25% trypsin (Gibco, USA) for 30 min and 0.2% type II collagenase (Sigma-Aldrich, USA) for 4 h at 37 °C with constant gentle agitation. The resulting single-cell suspension was filtered through a 70 μm cell strainer and centrifuged at 1,000 rpm for 5 min, then cultured in DMEM/F12 (Gibco, USA) supplemented with 10% FBS and 1% penicillin-streptomycin at 37 °C in a 5% CO₂ atmosphere. Passaged cells (P2) were used for subsequent experiments.

### 2.7 Western blotting

Total protein lysates from NP cells were extracted using RIPA lysis buffer supplemented with 1% PMSF and quantified via BCA assay (Beyotime, China). Equal amounts of protein (30 μg per lane) were separated by 8%–12% SDS-PAGE and transferred to PVDF membranes (Millipore, USA). Membranes were blocked with 5% non-fat milk in TBST (0.1% Tween-20 in Tris-buffered saline) for 1 h at room temperature (RT) and incubated overnight at 4 °C with primary antibodies against Lgr5 (Abcam, ab75850, rabbit polyclonal, 1:1,000),Collagen II (Abcam, ab34712, rabbit polyclonal, 1:1,000), MMP13 (Abcam, ab219620, rabbit polyclonal, 1:1,000), p-P65 (Abcam, ab76302, rabbit polyclonal, 1:1,000), and β-actin (Abcam, ab8227, mouse monoclonal, 1:5,000, loading control). After incubation with HRP-conjugated secondary antibodies (goat anti-rabbit IgG, 1:5,000; goat anti-mouse IgG, 1:5,000), signals were visualized using ECL substrate (Millipore, USA) and quantified densitometrically with ImageJ.

### 2.8 Immunohistochemistry

Paraffin-embedded sections were deparaffinized, rehydrated, and subjected to antigen retrieval by boiling in 0.01 M citrate buffer (pH 6.0) for 15 min. Endogenous peroxidase activity was quenched with 3% H_2_O_2_ for 10 min at RT, followed by blocking with 5% normal goat serum (Solarbio, China) for 30 min at RT. Sections were incubated overnight at 4 °C (approximately 16 h) with primary antibodies against MMP13 (Abcam, ab219620, rabbit polyclonal, 1:200), p-p65 (Abcam, ab76302, rabbit polyclonal, 1:200), Active caspase-3 (Abcam, ab179517, rabbit polyclonal, 1:200), and PCNA (Abcam, ab92552, rabbit polyclonal, 1:5,000), CTSK (Abcam, ab19027, rabbit polyclonal, 1:200), and c-Fos (Abcam, ab222699, rabbit monoclonal, 1:500). After secondary antibody incubation, signals were developed with 3,3′-diaminobenzidine (DAB) (Solarbio, China) and counterstained with hematoxylin. Negative controls were established by replacing primary antibodies with PBS. The integrated optical density (IOD) was measured using ImageJ from five random fields per section, and values were normalized to the number of Hematoxylin-stained nuclei per field. Only sections with intact tissue morphology were included. All quantifications were performed by two independent investigators blinded to genotype.

### 2.9 Immunofluorescence staining

Nucleus pulposus cells were seeded in confocal culture dishes (Nest, China) at a density of 5 × 10^4^ cells/mL. After 24 h of culture, cells were fixed with 4% PFA for 15 min, permeabilized with 0.1% Triton X-100 for 15 min, and blocked with 5% normal goat serum (Solarbio, China) for 30 min. Cells were incubated with primary antibodies against MMP13 (Abcam, ab219620, rabbit polyclonal, 1:100), p-P65 (Abcam, ab76302, rabbit polyclonal, 1:100), Active caspase-3 (Abcam, ab179517, rabbit polyclonal, 1:100), and PCNA (Abcam, ab92552, rabbit polyclonal, 1:200) overnight at 4 °C, followed by Alexa Fluor 594-conjugated fluorescent secondary antibodies (goat anti-rabbit IgG, 1:200) at RT for 1 h in the dark. Nuclei were stained with DAPI (Beyotime, China) for 5 min in the dark. Negative controls were established by replacing primary antibodies with PBS. Confocal images were acquired using an Olympus FV3000 system with fixed laser power (2% for 594 nm, 1% for 405 nm) and PMT gain (600 for both channels) across all samples. Fluorescence intensity was quantified using ImageJ and normalized to DAPI nuclear counts per field. Only fields with intact cells and no visible debris were analyzed. All quantifications were performed by two independent investigators blinded to genotype. Each experiment was repeated three times.

### 2.10 Statistical analysis

Data are presented as mean ± standard deviation (SD). Normality was assessed using the Shapiro–Wilk test. Statistical comparisons between two groups were performed using two-tailed unpaired Student's *t*-test in SPSS 26.0. For comparisons involving both genotype and surgery conditions, two-way ANOVA with Bonferroni *post-hoc* correction was used. For multiple comparisons, *P*-values were adjusted using the Benjamini-Hochberg method to control the false discovery rate (FDR). Sample sizes for each experiment were determined based on preliminary pilot studies or power analysis. A value of *P* < 0.05 was considered statistically significant. All experiments were performed in at least three independent replicates.

## 3 Results

### 3.1 Lgr5 deficiency impairs intervertebral disc integrity and trabecular microarchitecture in mice

First, we determined the cellular distribution of Lgr5 within the intervertebral disc by immunohistochemical staining using an anti-Lgr5 antibody on lumbar disc sections from 8-month-old wild-type mice. As shown in Supplementary Figure S1, Lgr5 immunoreactivity (brown) was predominantly detected in the nucleus pulposus (NP), with relatively weaker signals in the annulus fibrosus (AF) and cartilaginous endplate (CEP). Whereas, the Lgr5 immunoreactivity was almost not detectable in Lgr5 KO tissues. Then we checked the impact of Lgr5 deficiency on microarchitecture of intervertebral disc by micro-CT analysis of the caudal vertebra of Lgr5 KO and WT mice. The micro-CT imaging revealed distinct morphological differences between genotypes. WT mice displayed well-preserved intervertebral disc architecture characterized by uniform disc height and intact vertebral endplates. In contrast, Lgr5 KO mice exhibited significant narrowing of the disc space, and obvious deterioration of trabecular bone microarchitecture (Figures 1A,B). Quantitative analyses further verified significantly decreased disc height index and bone volume fraction (BV/TV) in Lgr5-deficient animals, indicating that loss of Lgr5 compromises both intervertebral disc structural integrity and subchondral bone quality (Figures 1C,D).

**Figure 1:**
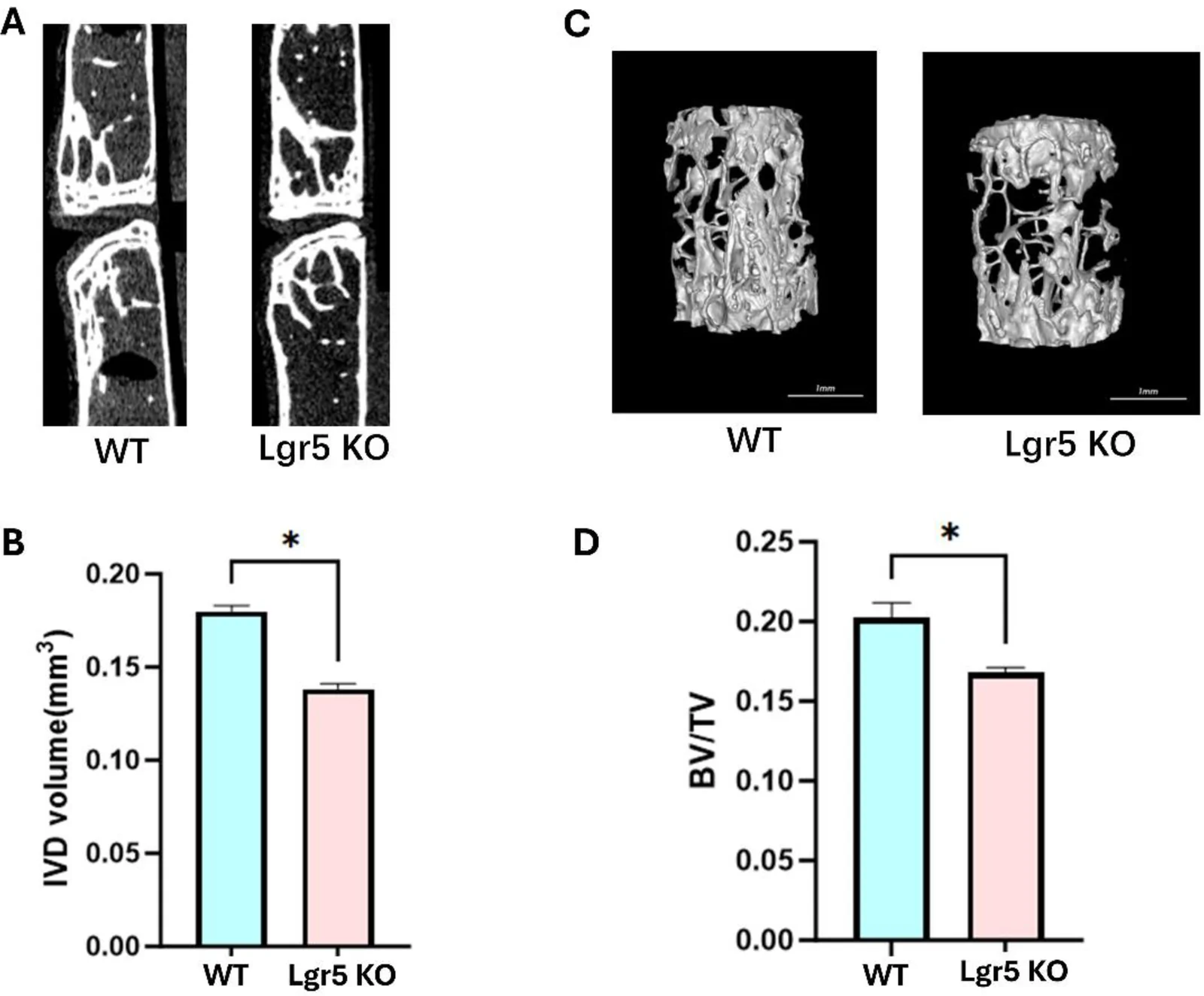
Micro-CT analysis of intervertebral discs of Lgr5 KO and WT mice. **(A)** Representative micro-CT images demonstrating significantly thinner intervertebral discs in the Lgr5 KO group compared to WT controls. **(B)** Quantitative analysis of intervertebral disc (IVD) volume. Data are presented as the mean ± SD. *P < 0.05, *n* = 5 per group. **(C)** Three-dimensional (3D) reconstructed images of trabecular bone illustrating microarchitectural differences between genotypes. **(D)** Quantitative analysis of trabecular bone volume fraction (BV/TV). Data are presented as the mean ± SD. *P < 0.05, *n* = 5 per group.

### 3.2 Lgr5 deficiency accelerated intervertebral disc degeneration

Next, we established the surgically induced intervertebral disc degeneration (IDD) model using Lgr5 KO and WT mice. Histological assessment corroborated the imaging findings. Hematoxylin and eosin (HE) staining showed that WT discs retained organized structure with abundant NP cells and a distinct NP-annulus fibrosus (AF) boundary, whereas Lgr5 KO discs displayed pronounced architectural disruption, NP atrophy, and cellular depletion. Safranin O/fast green (SFO) staining revealed robust glycosaminoglycan (GAG) content in WT endplates and NP, which was markedly diminished in knockout mice, indicating severe ECM degradation (Figure 2).

**Figure 2:**
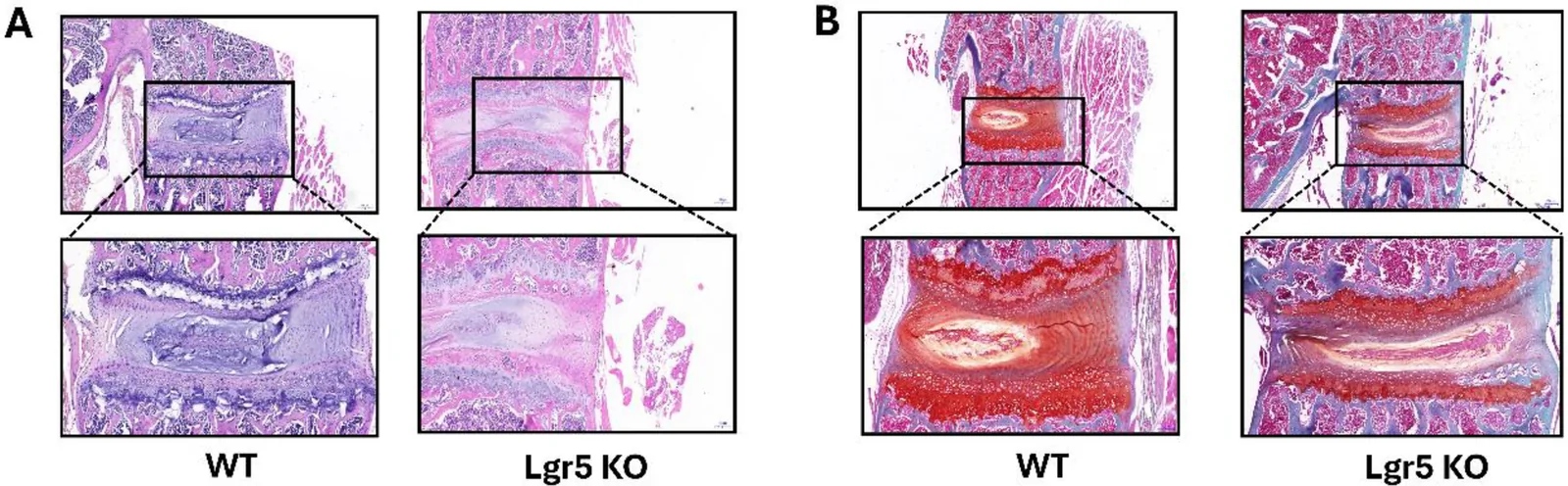
HE and SFO staining for assessing intervertebral disc degeneration. **(A)** HE staining showing disc structure. **(B)** SFO staining showing GAG distribution. Scale bar: 50 μm. *n* = 5 per group.

### 3.3 Transcriptomic landscape of intervertebral discs in Lgr5 KO mice

At 4 weeks post-surgery, we performed RNA-seq analysis on caudal vertebral discs tissues isolated from wild-type and Lgr5 KO mice. The Heatmap and volcano plot illustrating differential gene expression were presented in Figures 3A,B. It was found that 457 genes were upregulated and 443 genes were downregulated in Lgr5 KO intervertebral discs compared with WT groups. The KEGG pathway enrichment analysis revealed prominent activation of multiple signaling cascades in the discs of Lgr5-deficient mice, including osteoclast differentiation, extracellular matrix (ECM)-receptor interaction, Toll-like receptor signaling, and complement cascades (Figure 3C). GO enrichment analysis further demonstrated that the differentially expressed genes were mainly enriched in terms related to the extracellular region, collagen-containing ECM, and membrane-associated structures, suggesting that Lgr5 deletion primarily disrupts ECM organization, membrane-related signaling events, and matrix structural integrity (Figure 3D). Specifically, the ECM-receptor interaction-related genes, and NF-κB signaling-related genes were shown in Figures 3E,F.

**Figure 3:**
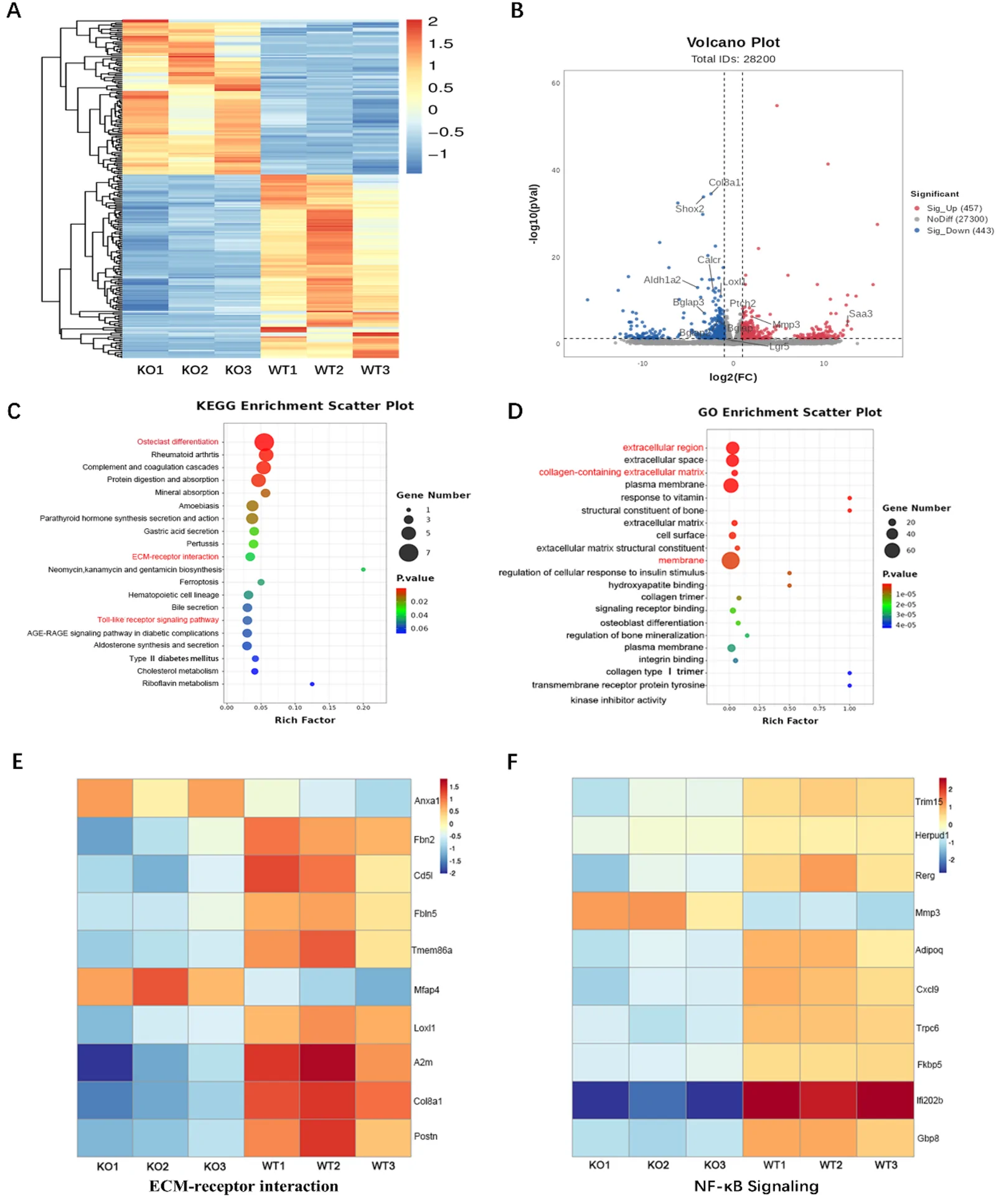
Transcriptomic profiling reveals activation of osteoclastogenic and inflammatory pathways in intervertebral discs of Lgr5 KO mice. **(A)** Heatmap of the top DEGs, showing upregulation of osteoclast-related, proinflammatory, and matrix-degrading genes, accompanied by downregulation of key extracellular matrix (ECM) structural components. **(B)** Volcano plot illustrating global gene expression changes. Red and blue dots represent significantly upregulated and downregulated genes, respectively (|log₂FC| ≥ 1, adjusted *P* < 0.05. **(C)** KEGG enrichment scatter plot displaying significantly enriched signaling pathways in Lgr5 KO disc tissues relative to WT controls. **(D)** GO enrichment analysis of biological processes associated with the identified differentially expressed genes (DEGs). **(E)** Heatmap of ECM-receptor interaction-related genes. **(F)** Heatmap of NF-κB signaling-related genes. (|log2FC| ≥ 1, adjusted *P* < 0.05).

### 3.4 Lgr5 deficiency activates osteoclastogenesis and inflammatory signaling in intervertebral discs

To further validate the findings of RNAseq analysis, the intervertebral discs were subjected to Tartrate-resistant acid phosphatase (TRAP) staining. We found significantly enhanced osteoclast activity in Lgr5 KO endplates, as evidenced by increased numbers of TRAP-positive cells and staining intensity compared to WT (Figures 4A,B). This was further corroborated by immunohistochemical staining for c-Fos and CTSK, two specific osteoclast differentiation markers, which showed markedly stronger positive signals in Lgr5 KO endplates (Figures 4C–F), confirming enhanced osteoclastogenesis at the molecular level. The western blot analysis performed on isolated nucleus pulposus (NP) cells demonstrated that Lgr5 deficiency significantly reduced Collagen II expression, while markedly up-regulated the protein levels of MMP13 and p-p65, relative to WT controls (Figure 5).

**Figure 4:**
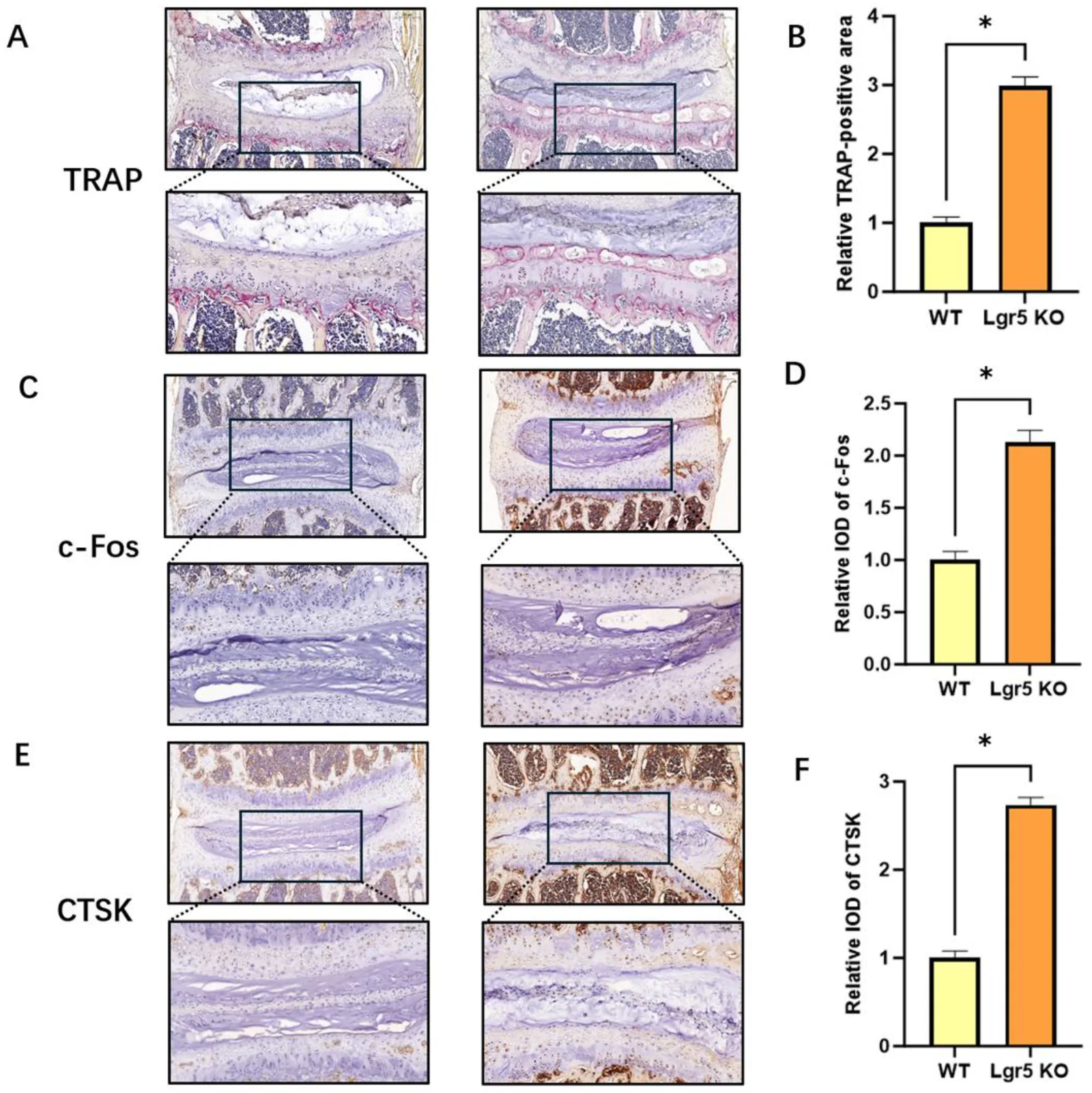
TRAP staining and osteoclast marker validation. **(A)** Representative images of TRAP staining (purplish-red multinucleated cells indicate positive osteoclasts). **(B)** Quantitative analysis of IOD in positive areas. **(C)** Representative IHC images of c-Fos staining in vertebral endplates. **(D)** Quantitative IOD analysis of c-Fos-positive areas. **(E)** Representative IHC images of CTSK staining. **(F)** Quantitative IOD analysis of CTSK-positive areas. Scale bar: 200 μm for TRAP and IHC. *P < 0.05, *n* = 5 per group.

**Figure 5:**
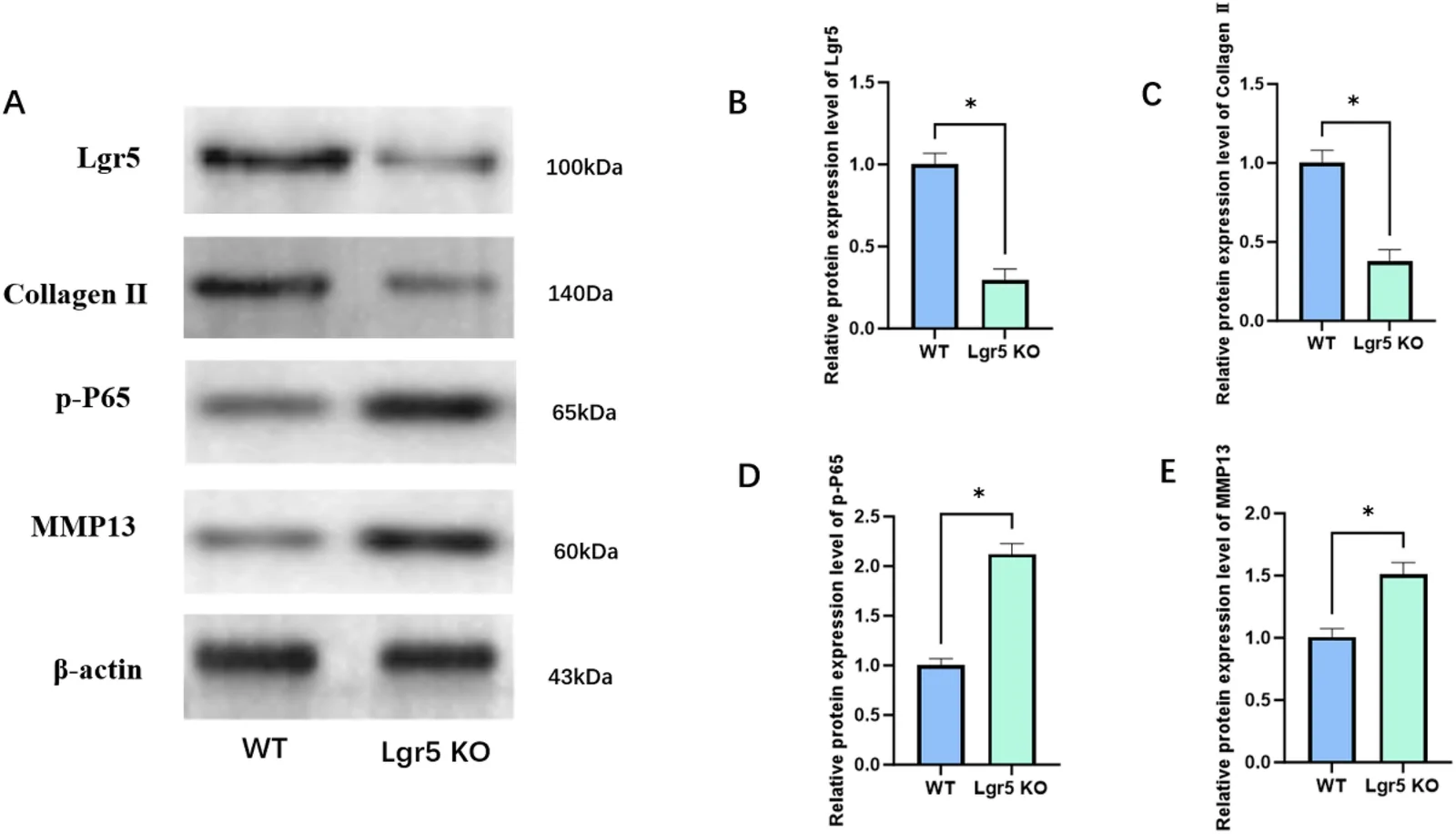
Western blot analysis of collagen II and inflammation pathway-related protein expression in nucleus pulposus cells. **(A)** Representative protein bands detected with indicated antibodies. **(B)** Relative expression of Lgr5. **(C)** Relative expression of Collagen II. **(D)** Relative expression of p-P65. **(E)** Relative expression of MMP13. *P < 0.05, *n* = 5 per group, β-actin served as the loading control.

### 3.5 Lgr5 deficiency promotes inflammation, apoptosis and suppresses proliferation in nucleus pulposus cells

In addition, Immunofluorescence staining of cultured NP cells demonstrated significantly higher fluorescence intensities for MMP13 and p-p65, along with reduced PCNA signal and elevated Active caspase 3 levels in Lgr5 KO cells relative to WT controls (Figure 6). This was further confirmed by Immunohistochemical staining of disc sections, showing markedly increased MMP13, p-p65, and Active caspase 3 in Lgr5 KO tissues, accompanied by a significant reduction in PCNA-positive cells (Figure 7). All quantitative data were normalized to Hematoxylin nuclear counts to correct for cell density variations.

**Figure 6:**
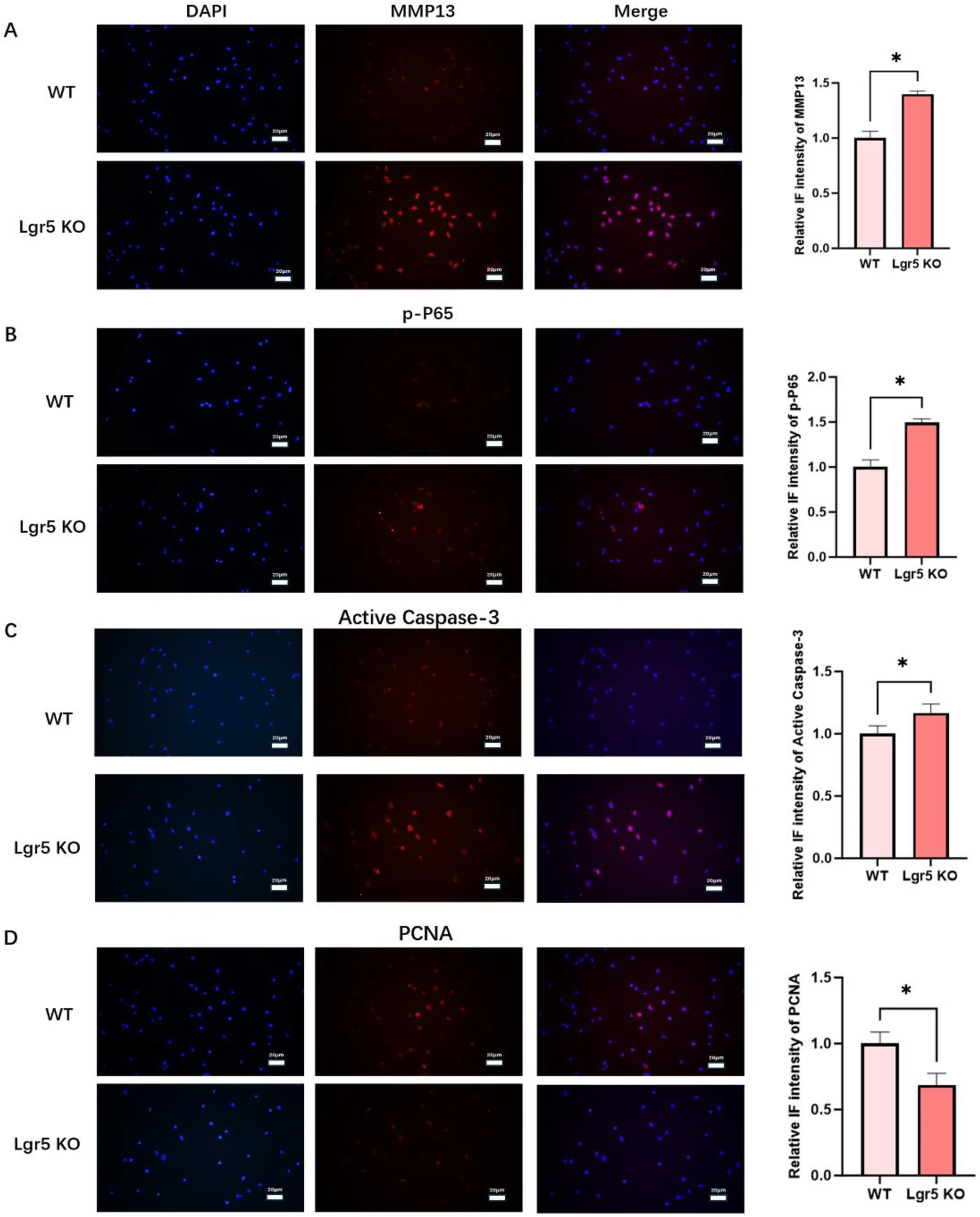
Immunofluorescence detection of protein expression in nucleus pulposus cells. Immunofluorescence staining was performed to detect the expression and subcellular localization of key functional proteins with indicated antibodies. **(A)** MMP13. **(B)** p-p65. **(C)** PCNA. **(D)** Active caspase-3. The nuclei were stained with DAPI. Scale bar: 20 μm. *P < 0.05, *n* = 5 per group.

**Figure 7:**
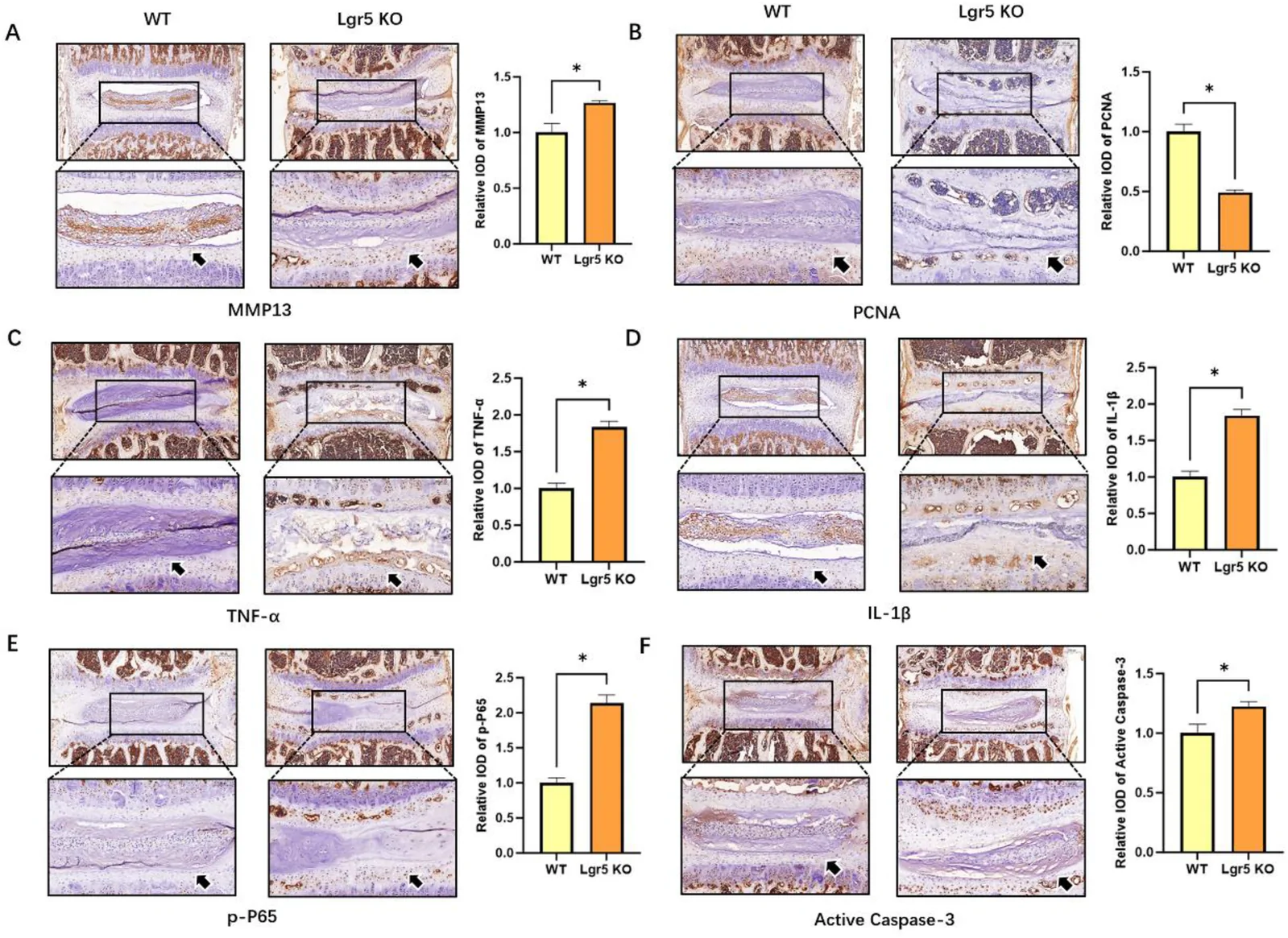
Immunohistochemical detection of protein expression in intervertebral disc tissue. Representative IHC images (brown color indicates positive staining) and IOD analysis. **(A)** MMP13. **(B)** PCNA. **(C)** TNF-α. **(D)** IL-1β. **(E)** p-P65. **(F)** Active Caspase-3. Scale bar: 200 μm. *P < 0.05, *n* = 5 per group.

## 4 Discussion

IDD represents a complex, multifactorial degenerative process orchestrated by interdependent pathological events, including ECM catabolism, cellular apoptosis, inflammatory signaling, and endplate dysfunction (18). In the present study, we found that Lgr5 deficiency was associated with accelerated IDD through the coordinated activation of NF-κB signaling and enhanced osteoclastogenesis, which finally led to disruption of NP cell homeostasis. These findings advance our understanding of the molecular circuitry governing disc degeneration and identify Lgr5 as a potential therapeutic target for IDD that merits further mechanistic validation.

As a facilitator of Wnt/β-catenin signaling, Lgr5 is instrumental in tissue homeostasis and stem cell regulation (19). Our data demonstrate that Lgr5 loss triggers profound structural deterioration, characterized by NP atrophy, GAG depletion, and downregulation of Collagen II (20). The concomitant upregulation of MMP13—a potent collagenase targeting Collagen II and aggrecan—implicates Lgr5 in restraining catabolic enzyme expression (21). These results align with the concept that Lgr5-mediated signals preserve ECM integrity by antagonizing pathways that drive matrix degradation in intervertebral discs.

Transcriptomic profiling revealed the enrichment of NF-κB pathway, which promotes transcription of inflammatory cytokines and MMPs while inducing apoptosis via executioner caspases (22). The elevated caspase 3 and diminished PCNA signals in Lgr5 KO NP cells provide direct evidence that Lgr5 loss shifts the balance from cell survival toward apoptosis and growth arrest. The increased IL-1β and TNF-α levels detected in Lgr5 KO disc tissues further support the notion that Lgr5 deficiency fosters a pro-inflammatory microenvironment,perpetuating a deleterious cycle of inflammation, cell death, and matrix degradation (23). Beyond its direct effects on NP cell homeostasis, Lgr5 deficiency also influences the microenvironment of the disc by modulating endplate remodeling (24).

The cartilaginous endplate serves as the primary nutrient gateway for the avascular disc, and its degeneration is a critical accelerator of IDD (25). Our observation of heightened TRAP activity increased CTSK and c-Fos expressionin in Lgr5 KO mice indicates that Lgr5 normally suppresses osteoclastogenesis. Enhanced osteoclast activity likely disrupts endplate integrity, impairs nutrient diffusion to NP cells, and exacerbates their metabolic stress (26). The concomitant reduction in BV/TV and trabecular disorganization observed by micro-CT aligns with this pathological bone remodeling. Collectively, these findings suggest that Lgr5 protects against IDD in part by restraining osteoclast-mediated endplate degradation (27). This is consistent with emerging evidence that LGR family members play important roles in skeletal tissues, where they regulate stem cell populations and bone homeostasis in the vertebral column.

While this study provides novel insights into Lgr5 function in IDD, several limitations warrant consideration (28). The mechanistic link between Lgr5 loss and pathway activation remains correlative; direct regulatory relationships have not been established using pharmacological inhibitors or genetic approaches. And the present study is based on a mouse model, and the translational value of these findings to human IDD needs to be further verified using clinical samples and human cell models.

### 4.1 Study limitations

Several limitations of this study warrant consideration. First, the Prx1-Cre driver used for conditional Lgr5 knockout targets broad mesenchymal lineages rather than NP- or endplate-specific cells. Although we confirmed Lgr5 expression in NP cells of wild-type mice, off-target deletion in other skeletal tissues may contribute to the observed phenotypes. Future studies using NP-specific (e.g., Shox2-Cre) or cartilage-specific (e.g., Col2a1-CreER) Cre lines are needed to validate cell-autonomous effects. Second, the mechanistic link between Lgr5 loss and NF-κB pathway activation remains correlative, as we did not perform rescue experiments with NF-κB inhibitors (e.g., BAY 11-7082) or Lgr5 overexpression in NP cells. Third, our findings are based solely on a mouse model; the translational relevance to human IDD requires verification using clinical disc samples and human NP cell culture systems. Fourth, we only assessed a single 8-week post-surgery time point, missing the dynamic temporal progression of IDD. A time-course analysis (e.g., 2, 4, 8, 12 weeks post-surgery) would better characterize the evolution of degenerative changes. Addressing these limitations in future investigations will be critical to consolidate Lgr5 as a therapeutic target for IDD.

## 5 Conclusion

This study demonstrates that Lgr5 deficiency is associated with accelerates IDD correlating with NF-κB signaling, promotion of osteoclast-mediated endplate remodeling, induction of NP cell apoptosis, suppression of NP cell proliferation, and exacerbation of ECM degradation. By integrating these pathogenic processes, Lgr5 emerges as a critical endogenous protector of disc homeostasis and a promising therapeutic target for slowing IDD progression.

## Data Availability

The datasets presented in this study can be found in online repositories. The names of the repository/repositories and accession number(s) can be found below: https://ngdc.cncb.ac.cn/gsa/s/YeQQ0nOw, CRA041529.
