# Supplementary material for: Lgr5 deficiency accelerates intervertebral disc degeneration via NF-κB activation and enhanced osteoclastogenesis
Source: Front Med (Lausanne). 2026 Oct 14;13:1939627. doi: 10.3389/fmed.2026.1939627 (PMC13713331; doi:10.3389/fmed.2026.1939627)
Supplement: Supplementary file 1 [file Table1.docx]

**Supplementary Fig. 1. Localization of Lgr5 expression in mouse intervertebral disc by immunohistochemistry.** Immunohistochemical staining of lumbar disc sections from 8-month-old wild-type mice using anti-Lgr5 antibody (brown). Nuclei were counterstained with hematoxylin (blue). Lgr5 immunoreactivity was predominantly detected in the nucleus pulposus (NP), with weaker expression in the annulus fibrosus (AF) and cartilaginous endplate (CEP). Scale bar: 200 μm. n = 3.
